# Complete Genome Sequence of Strain H5989 of a Novel *Devosia* Species

**DOI:** 10.1128/genomeA.00934-15

**Published:** 2015-09-03

**Authors:** Ainsley C. Nicholson, Anne M. Whitney, Ben Humrighouse, Brian Emery, Vladimir Loparev, John R. McQuiston

**Affiliations:** a Special Bacteriology Reference Laboratory, Bacterial Special Pathogens Branch, Division of High Consequence Pathogens and Pathology, Centers for Disease Control and Prevention, Atlanta, Georgia, USA; b Division of Scientific Resources, Centers for Disease Control and Prevention, Atlanta, Georgia, USA

## Abstract

The CDC Special Bacteriology Reference Laboratory (SBRL) collection of human clinical pathogens contains several strains from the genus *Devosia*, usually found environmentally. We provide here the complete genome of strain H5989, which was isolated from a human cerebrospinal fluid (CSF) specimen and represents a putative novel species in the genus *Devosia*.

## GENOME ANNOUNCEMENT

Strain H5989 was derived from a human clinical cerebrospinal fluid (CSF) specimen and was characterized as a Gram-negative fermentative rod. Results of 16S rRNA gene sequence analysis were consistent with the isolate being a member of the *Hyphomicrobiaceae* family and were 99.1% identical to those for the 16S rRNA gene from strain J5-3 of the recently described species *Paradevosia shaoguanensis*. Calculation of the percentage of conserved proteins (POCP) using the method described by Qin et al. (1) showed strain H5989 to have between 67% and 74% POCP compared to whole-genome sequences of nine *Devosia* species. This analysis taxonomically places H5989 among the *Devosia* species and suggests that the *Paradevosia* strain may also belong to the genus *Devosia*.

Strain H5989 was grown at 35°C in a candle jar on heart infusion agar with 5% rabbit blood. Genomic DNA was purified using the Department of Energy Joint Genome Institute’s phenol-chloroform-cetyltrimethylammonium bromide (CTAB) extraction procedure according to the directions available online (2). Genome sequencing libraries were prepared and sequence reads generated on the Illumina MiSeq platform using Illumina TruSeq chemistry and following the manufacturer’s instructions (Illumina, Inc., San Diego, CA). Reads were trimmed based on quality (limit, 0.05), and the resulting 2,147,784 reads (averaging 151 bp) were assembled using the De Bruijn graph method of *de novo* assembly provided by CLC Genomics Workbench version 7.51.

The initial assembly yielded 24 contigs of at least 500 bp, with approximately 65× coverage. These contigs were oriented to each other using a KpnI optical map (OpGen, Gaithersburg, MD). Reads corresponding to the ends of each adjacent contig were located, sorted, and positionally aligned using JMP (JMP, version 10; SAS Institute, Inc., Cary, NC) and then visualized using BioEdit (3). Contigs were joined based on evidence from the read sequence alignments, and ultimately a single circular 4.6-Mbp chromosome was deduced, which had a predicted restriction map consistent with the optical map. A total of 95.8% of the trimmed reads mapped back to this chromosome sequence, and *de novo* assembly using the unmapped reads yielded no contigs longer than 500 bp.

The completed genome was automatically annotated by the PGAP pathway at NCBI and the use of the RAST server (4, 5), and both annotations were compared with KEGG annotations of open reading frames (6). The final annotation consisted of 4,296 protein-encoding regions, 47 tRNAs, and 2 rRNA operons.

### Nucleotide sequence accession numbers.

The complete genome sequence of H5989 has been deposited at GenBank under the accession number CP011300, BioProject number PRJNA281416, and BioSample accession number SAMN03487588.
